# Characterizing forensically important insect and microbial community colonization patterns in buried remains

**DOI:** 10.1038/s41598-018-33794-0

**Published:** 2018-10-19

**Authors:** Lavinia Iancu, Emily N. Junkins, Georgiana Necula-Petrareanu, Cristina Purcarea

**Affiliations:** 1 Institute of Biology Bucharest, Romanian Academy, Splaiul Independentei, 296, 060031 Bucharest, Romania; 2 University of Oklahoma, Department of Microbiology and Plant Biology, 770 Van Vleet Oval, Norman, OK 73019-0390 United States of America

## Abstract

During violent criminal actions in which the perpetrator disposes of the victim’s remains by burial, the analysis of insects and bacterial colonization patterns could be necessary for postmortem interval (PMI) estimation. Our research aimed to assess the decomposition process of buried rat carcasses from shallow graves (40 cm), the diversity and dynamics of insects and bacteria throughout the decomposition stages, and the environmental parameters’ influence on these variations. The results provide further insight on decomposition in soil and contribute to a broader understanding of the factors involved in decomposition by qualitatively and quantitatively analysing the decomposer community (bacteria and insects). Additionally, two bacterial taxa, *Enterococcus faecalis* and *Clostridium paraputrificum* that were investigated for the first time as PMI indicators using quantitative polymerase chain reaction (qPCR) showed differential abundance over time, promising data for PMI estimation. The current study on the decomposition of buried rat carcasses in a natural environment will strengthen the current knowledge on decomposed remains from shallow graves and represents an effort to quantify insect and bacterial taxa as PMI estimators.

## Introduction

In many violent criminal cases, a perpetrator tends to conceal a crime and dispose of a victim’s remains by burial, often digging shallow graves, due to the size and weight of a human body^1^. Buried remains are thus exposed to different factors that affect decomposition and overall the postmortem interval (PMI) estimation. Such factors can be abiotic (soil pH, soil temperature, soil type and moisture) or biotic (environmental microbial communities and insect colonizer accessibility)^2^. Considering the biotic factors influence, microbial and insect dynamics during decomposition are important factors when estimating the PMI for buried remains. However, most of the experimental research regarding the insect and bacterial species associated with the decomposition process have been investigated using surface remains^3–11^. Because of this, the current study aimed to investigate the decomposition process of buried rat carcasses (*Rattus norvegicus*, Berkenhout 1769) as analogues for human cadavers.

Several other investigations on the decomposition process of buried remains have been reported from Great Britain^12–17^ Poland^18^, Australia^19^, Argentina^20^, United States of America^21–25^, Canada^26,27^, Germany^28^, and South Africa^29^. Most of them used human cadavers^17,20–23,26,27^ and pig carcasses^13,14,18,24,25^, few used juvenile rat^19^ and sheep carcasses^12^ as experimental models, but lacked the combined bacterial and insect community analyses. Overall, these studies focused on different aspects of decomposition starting with preliminary observations of the decay process within different soil types^19^, necrophagous insect species colonization patterns^13,26,30^ and, more recently, characterization of the decomposition necrobiome structure^31,32^ for forensic application.

Carcass decomposition in soil differs from the surface remains. Surface remains often exhibit distinct stages of decomposition: fresh, bloat, active decay, advanced decay and skeletal remains^33^. Like surface remains, buried remains can exhibit defined stages of decomposition^34^, beginning with fresh stage, which continues until the swelling of the abdomen is visible, followed by primary and secondary bloat stage, characterized by accumulation of gasses and strong odour. As the abdomen deflates, the remains transition into the active decay where tissues are still present, with decomposer and scavenger communities active. As the remains are utilized and begin to dry, decomposition transitions to the advanced decay stage, during which the abdomen and rib cage collapse, flesh is liquefied or absent, and often adipocere formation can be observed as the remains transition into the skeletal stage^34^. Moreover, in the case of both surface and buried remains, the decomposition process is strongly affected by taphonomic factors such as ante mortem conditions (covering or clothes and wounds), environmental factors like grave and soil characteristics, season and temperature, and insect and scavenger access^35^.

Of these factors, temperature variation plays an important role in carcasses decomposition, with the range of 20–40 °C optimal for tissue degradation^29^, particularly 37 °C^36^. Temperatures below 10 °C and over 40 °C will potentially inhibit decomposition^30^. Additionally, changes in climate fluctuations affect insect activity and influence the possibility of carcass colonization in soil. The decomposition process in buried environments is also dependent on the oxygen content, which in small quantities can affect the microbial community composition and structure during decomposition, while slowing down the process due to its effect on insect colonization^30^. In addition to the environmental variables (depth, geographical location, season, meteorological conditions) that must be considered when investigating a buried location, the soil composition and chemistry is another important factor for decomposition that must be considered^13^.

Further, the soil necrophagous entomofauna is also very different from that observed and identified from surface remains, as dominant families differ in number and diversity. Frequently, Phoridae and Muscidae specimens are collected from buried carcasses^14^, unlike Calliphoridae family representatives that comprise the primary and dominant colonizers of surface remains. Ultimately, the colonization process takes place when the necrophagous insects lay eggs on the soil surface or crawl through the soil matrix to reach the carcass. This behaviour can be influenced by season, temperature, soil granularity and depth of burial^14^.

So far studies reported Phoridae, Muscidae and Sarcophagidae as dominant species recovered from buried remains^30,37–39^. Case reports describing necrophagous entomofauna from buried bodies are scarce as most are directed towards exhumation studies^20^.

Furthermore, the emerging field of forensic microbiology has attempted to solve certain problems encountered when estimating postmortem interval by using predictable changes in the microbial community structure. These studies focus on the necrobiome structure from vertebrate carcasses^31,40^, on the microbial communities shifts during the decomposition process^41–45^, and on the characterization of soil microbial and fungal communities associated with the carcass^32,46,47^. Overall, forensic microbiology also seeks to complement forensic entomology by providing another tool for postmortem interval estimation by following the same principles of forensic entomology. However, studies investigating the microbiome of decomposition and the insect microbiome focus mostly on surface remains^11,31,40–44,48,49^, while fewer studies focused on buried remains. Though these investigations were foundational to elucidating the decomposition process, they were either performed under laboratory-controlled conditions, or they only focused on the diversity of soil-associated bacterial communities^10,44,46,47^.

Our current understanding shows that soil, host-associated, and insect-associated microbial communities change over time as decomposition progresses. Since the forensic interest lies in an investigator’s ability to track these changes overtime, many research endeavours focus on determining these overall changes in the community overtime, but also on specific changes in certain members of these communities with the hope that they could be used as postmortem interval estimators. A study that focused on specific taxa of the human gut microbiome found that over the course of decomposition, certain taxa could be tracked over time. Specifically, *Bacteroides* sp., *Lactobacillus* sp. and *Bifidobacterium* sp. were quantified via qPCR during the decomposition of human cadavers as putative quantitative indicators of PMI^50^. Other studies concerning phylum Firmicutes quantitative analysis have been focused on analysing water samples^51^, or the overall intestinal microbiota^52,53^.

To further understand the role of insects and microbes in the decomposition process of buried remains and how it pertains to the postmortem interval estimation, the present research provides a complex insight into both insects and bacteria as primary decomposers and environmental factors that could influence the process. To observe changes in insect dynamics and the microbial community, rat carcasses were used as analogues for human remains. First, rat carcasses are widely used as animal models in clinical human studies^54–59^, generating a level of replication and destructive sampling that is easier to achieve compared to human samples. Also, rodents have been successfully used in previous forensic experiments^45,60^, as the decomposition process and microbial composition of a rat carcass is comparable to a human cadaver^45^. The experiment was performed during two periods of 30 days in spring and summer (March and June) in a temperate continental climate zone of Eastern Europe (Bucharest, Romania). The main objectives of this study were to qualitatively and quantitatively asses the necrophagous entomofauna and the necrobiome throughout the decomposition process using buried rat carcasses, and to investigate the environmental factors affecting the decomposition process, describing at the same time the colonization patterns of the main decomposers. Understanding the necrophagous insect species colonization behaviour and the carcass and insect-associated bacterial communities is significant when trying to determine PMI estimators for buried remains.

Lastly, this article provides the first postmortem quantitative data on *Enterococcus faecalis* and *Clostridium paraputrificum* (Firmicutes) identified from the proximal region of the rat small intestine and their potential use as putative indicators for the postmortem interval estimation.

## Material and Methods

### Environmental parameters and experimental design

The experimental site was situated in a private fenced green urban area of Bucharest, Romania (4482701000 N 2680500400 E), inhabited by trees (*Aesculus hyppocastanum*, *Tilia* sp., *Fraxinus* sp.), shrubs (*Buxus sempervirens*) and soil vegetation (*Ficaria verna*, *Elymus* sp.), while the soil type was Chernozems fine grained^61^. The experimental area was arranged along a perimeter of nearly 20 meters.

The decomposition survey took place in the spring (March) and summer (June) months of 2016, using a total of 60 adult rat carcasses (*Rattus norvegicus*, Berkenhout 1769) aged 56–84 days, that had comparable weights, and were fed the same diet. The specimens were euthanatized by carbon dioxide inhalation in accordance with the international and national legislation (EU Directive 86–609-EEC 2010/63/EU and law no. 43/2014), approved by the ethical committee of the Institute of Biology Bucharest where these analyses were performed. The carcasses were placed in shallow graves (40 cm depth) at a distance greater than 0.5 m, under natural environmental conditions (i.e. no coverings or restrictive boundaries). Rat carcasses were used as human analogues since they allowed a higher number of replicates and represent a widely used animal model for human biological research^62^. Moreover, rat carcasses have been used successfully in previous decomposition studies as experimental models^45,60^.

The air, soil temperature and relative humidity were recorded every 4 h using three button logger thermo-hygrometers (Ecotone, Gdynia, Poland), while the precipitation rate was obtained from the website of a National Meteorological Administration^63^ weather station located 8.5 km from the experimental site. Soil pH was measured daily in triplicate with a Portable pH/EC/TDS/Temperature Meter (Hanna Instruments, Woonsocket, RI, USA). Control sites were represented by empty graves from which pH measurements and insect presence were monitored in the absence of rat carcasses at the same depth (40 cm). No exposed carcasses were used as burial controls since the goal of this study was to describe taxa observed on buried remains and quantify taxa associated with changes in the gut communities of buried rat carcasses.

Each rat carcass was carefully excavated with a short-handle wood spade. The specimens were destructively sampled in triplicate, by removing a proximal part of the small intestine that was preserved in 200 µl Tris-EDTA pH 8 (TE) buffer at −20 °C, each carcass being discarded afterward. Thirty rat carcasses were used for each month. Necrophagous insect species (adults and larvae) were observed and collected daily, only in June, using entomological net and forceps. Specimens were preserved in 200 µl Tris-EDTA pH 8 (TE) buffer at −20 °C for DNA extraction, and in 75% ethanol for taxonomic identification. During each sampling time, larvae were placed in rearing jars, though none survived to pupate. Pupae were also collected from the rat carcasses and were kept at constant temperature and humidity until hatching. Given the low temperatures in March, no insects were collected.

### Necrophagous insect species taxonomic and genetic identification

The adult and immature stage insects were taxonomically identified with a Stereomicroscope Leica S6D (Leica Microsystems, Wetzlar, Germany) using the identification keys provided by Disney^64^, Gregor *et al*.^65^ and Grzywacz *et al*.^66^.

DNA barcoding was performed via the amplified insect COI (cytochrome c oxidase I) gene fragment^67^. Prior to DNA extraction, an additional step of cell disruption with 2 mm ZR Bashing Beads (Zymo research, Irvine, CA, USA) was performed, by using a SpeedMill PLUS Cell Homogenizer (Analitik Jena, Jena, Germany), at 20 °C, 50 Hz-power, for 12 min. Total DNA was extracted according with the manufacturer protocol from DNeasy Blood & Tissue Kit (Qiagen, Valencia, CA, USA). Following extraction, the DNA purity and concentration was measured with a Biodrop spectrophotometer (Denville Scientific Inc, Holliston, MA, USA) and amplified using a Mastercycler ProS System (Eppendorf, Wien, Austria). The PCR was performed in a total volume of 50 µl, containing 200 ng/µl insect DNA, 10 pmol/ml of forward LCO1490 and reverse HC02198 primers^68^, 0.1 mM dNTP, 1U of Taq DNA polymerase, and 1x Taq buffer of 2.5 mM MgCl_2_ (ThermoFisher Scientific, Waltham, MA, USA). The DNA amplification program comprised: 1 min at 94 °C, 5 cycles of 30 s at 94 °C (denaturation), 1.30 min at 45 °C (annealing), and 1 min at 72 °C (extension), 35 cycles of 30 s at 94 °C, 1.30 min at 51 °C, 1 min at 72 °C, and 5 min at 72 °C. The resulting DNA amplicons (710 bp) were analysed on 1% agarose gels (Cleaver Scientific, Rugby, UK), in the presence of a negative control. The PCR products were purified with PureLink PCR Purification Kit (ThermoFisher Scientific) and sent for sequencing to Macrogen Genomics (Netherlands).

The insect COI nucleotide sequences coverage and identity was verified with BLAST-NCBI^69^. Quality trimming and alignment of sequences was performed with Sequence Assembly and Alignment Software – CodonCode^70^. The identified sequences were deposited in the GenBank database with the accession numbers: [KY582492, KY582507, KY582509] for *Muscina prolapsa* third instar larvae, adult and teneral specimen (Diptera: Muscidae); [KY582497] for *Muscina levida* third instar larvae (Diptera: Muscidae); [KY582504] for *Hydrotaea ignava* third instar larvae (Diptera: Muscidae), found in Genbank as *leucostoma*; [KY582493, KY582496] for *Megaselia rufipes* (Diptera: Phoridae) larvae and adult; [KY582506] for Leiodidae sp. (Coleoptera) adult; and [KY582503] for *Medon brunneus* (Coleoptera: Staphylinidae).

### Illumina 16S rRNA gene sequencing for bacterial communities

Total DNA from rat tissue and insects was extracted according to the protocol described previously, while the DNA concentration was measured with Qubit fluorometer (ThermoFisher Scientific). PCR amplification of 16S rRNA gene fragments (V3-V4 variable region) was performed using the primer pair 341F/806R^71^ (McGill University and Génome Québec Innovation Centre, Canada). The barcoded amplicons were analysed in triplicate, pooled in equal concentrations and sequenced using one lane of the Illumina MiSeq PE300 sequencing platform at the McGill University and Génome Québec Innovation Centre.

Amplicon 16S rRNA sequences were quality filtered with a q-score of 30 and paired-end reads were merged in PEAR (v0.9.5) using a cut off length of 300 base pairs^72^.

Remaining sequences were de-replicated and singletons were filtered out using USEARCH^73^ via Quantitative Insights into Microbial Ecology (QIIME)^74^. Sequences were clustered via UPARSE^73^ into operational taxonomic units (OTUs). Each OTU was represented by the most abundant sequence in the cluster and was used as the reference database to which the original sequences were aligned to with 97% similarity and from which taxonomy was assigned using the QIIME formatted SILVA database^75^. The sequencing depth varied between 188 and 235,295 sequences per sample with a mean and median of 137,471 and 141,646 sequences, respectively. One triplicate from one sample (IP 14.2) was removed from the final dataset as it had the lowest sequencing depth (188 sequences) and was not assigned taxonomy. The final dataset consisted of a total of 2,049 OTUs from 69 samples. This data represents the complete dataset for this decomposition experiment. A subset of this data was used to describe a previously unobserved necrophagous insect in Romania^76^.

### Quantitative polymerase chain reaction (qPCR)

The relative abundances of *Enterococcus faecalis* and *Clostridium paraputrificum* during decomposition were determined by qPCR using specific primers for *E*. *faecalis* ENTFRev/ENTFFw^51^, and *C*. *paraputrificum* CLPARev/CLPAFw^77^. Thermo Scientific Maxima SYBR Green qPCR Master Mix (2x) (ThermoFisher Scientific) was used in a 10 µl reaction volume containing 0.3 µM of each forward and reverse primer, 200 ng template DNA and nuclease free water.

Amplification reactions were carried out in a Mastercycler ep gradient S thermocycler PCR (Eppendorf), by incubation for 10 min at 95 °C, followed by 40 cycles of 15 sec at 95 °C, 30 sec at 55 °C for *E*. *faecalis* or 56 °C for *C*. *paraputrificum*, and 30 sec at 72 °C. A melting curve analysis was included at the end of every program to ensure that the SYBR green assay produced the intended amplicon. Quantification was performed by interpolation in a standard regression curve of cycle threshold (Ct) values generated from samples of known concentration of DNA template. The PCR product itself amplified from a previous reaction, purified from the agarose gel (1%) and quantified by BiodropDuo UV/VIS Spectrophotometer (Harvard Bioscience Inc, Holliston, MA, USA), was used as template for the standard curve. A series of five decimal dilutions of DNA concentrations were used for standard between 3.7–3.7 × 10^−4^ ng/uL DNA for PCR product of *E*. *faecalis* and 3.9–3.9 × 10^−4^ ng/uL for PCR product of *C*. *paraputrificum*. The standard curves were plotted as C_t_ versus log_10_ (ng DNA/reaction) and considered when R^2^ > 0.99.

Real-time PCR assays with Ct values over 40 were considered negative. Non-template controls were included in all PCR runs and tested negative. Samples were analysed in triplicate, with two amplification runs for each sample.

Genome copies of DNA were calculated by the formulas previously used by Clifford and collaborators^78^. The size of genomes used were 3.2 × 10^6^ bp for *E*. *faecalis* (NCBI_Ref_Seq NC_004668.1) and 3.5 × 10^6^ bp for *C*. *paraputrificum* (NCBI_Ref_Seq NZ_AUJC01000001.1).

The results were shown as logarithm (base 10) of number of genomes per reaction versus postmortem days. Data were analysed using MS Excel.

### Data analyses and visualization

Statistical analyses were performed at a confidence level α = 0.05, using MS Excel and Past v3.04^79^. To investigate the influence of the environmental variables on the progress of the decomposition process during March and June, a principal component analysis (PCA) was performed. All variables were normalized prior to running the PCA. To determine if pH was correlated with the microbial diversity of each sample, a correlation analysis with Pearson’s r was run with the pH and Shannon diversity index in PRISM (v7.0).

To determine differences within samples, alpha diversity was calculated using an unweighted measure of observed OTUs and a weighted measure with Shannon’s Diversity Index in QIIME and PRISM.

Beta diversity analysis was used to compare community structure differences between samples. Principal coordinates analyses were constructed in QIIME based on weighted UniFrac distances. To observe how bacterial communities changed overtime and how bacterial abundance differed among samples, taxon relative abundances were calculated and visualized in PRISM (v7).

## Results

### The environmental conditions and decomposition process

#### Environmental conditions

**Figure 1:**
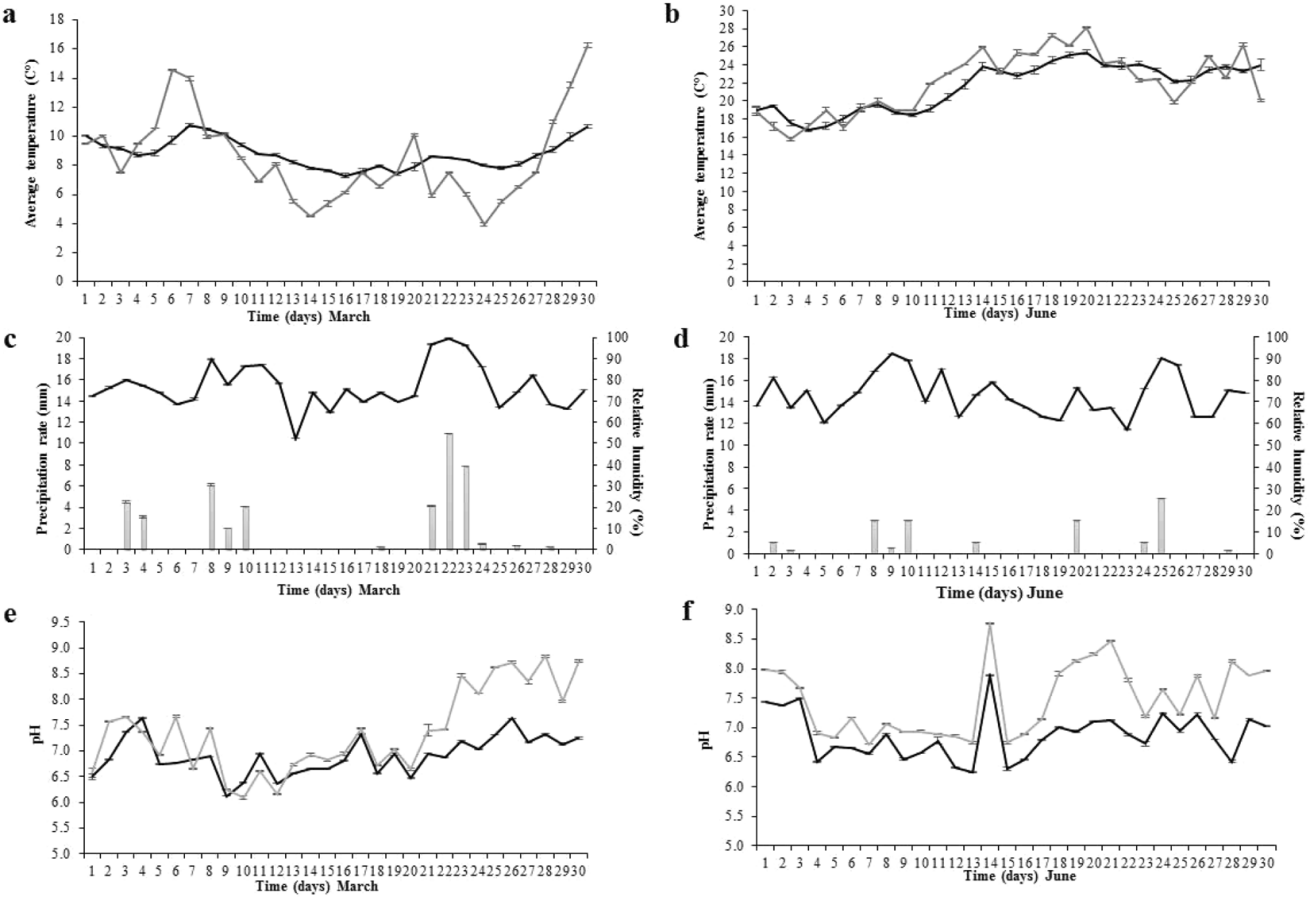
Environmental conditions during the experiment. Average temperature variation: (**a**) March; (**b**) June (soil - gray line; air - black line). Relative humidity and precipitation rate variation: (**c**) March; (**d**) June (relative humidity - black line; precipitation rate - gray bars); Soil pH variation during the investigated period: (**e**) March; (**f**) June (surrounding control soil pH – black line; grave soil pH – grey line). Standard deviations indicated by error bars (*n* = 3).

The air, soil temperature and relative humidity were recorded with three button loggers, while the soil pH was measured in triplicate. Averages with standard errors values were calculated (Fig. 1).

In March, the maximum soil and air temperature reached 10.7 °C and 16.3 °C, respectively, while 7.28 °C and 3.96 °C represented the minimum threshold (Fig. 1a). The difference between air and soil temperature varied from 0.04 °C up to 5 °C in the last experimental day. For June, the soil temperature ranged between 16.75 °C and 25.37 °C, while the air temperature varied between 15.8 °C and 28.13 °C (Fig. 1b). In this case, the differences between the two records were much lower within the range of 0.21–2.89 °C.

The air relative humidity ranged between 52.36–99.63% in March (Fig. 1c), and 57.13–92.2% in June (Fig. 1d) and coincided with the rainfall. March had a higher precipitation rate, reaching 10.91 mm, compared to June, with levels of 5.08 mm (Fig. 1c,d).

In March, the soil pH ranged from 6.0 to 7.6 for both surrounding soils, used as a control, and grave soil until day 20 where grave soil pH increased over time to above 8.0 on day 26, while the surrounding soil remained ranged between 6.8 to 7.4 (Fig. 1e). This pH increase in the grave soil corresponded with the active and advanced decay, though the delineation between these two stages was not clear.

In June, the changes in pH overtime were similar for both surrounding soil and grave soil, with the grave soil being slightly more alkaline (Fig. 1f). Unlike March, June saw more drastic changes in pH overtime. During the fresh stage, the pH was higher for both soils, after which a decrease was observed during the bloat stage, when the pH became more acidic, dropping to a value of 6.7 for the grave soil and 6.4 for the surrounding soil. A large increase in pH was observed for both the surrounding soil and the grave soil that corresponded with active decay (day 14) and advanced decay (day 18–21).

#### Decomposition process

**Figure 2:**
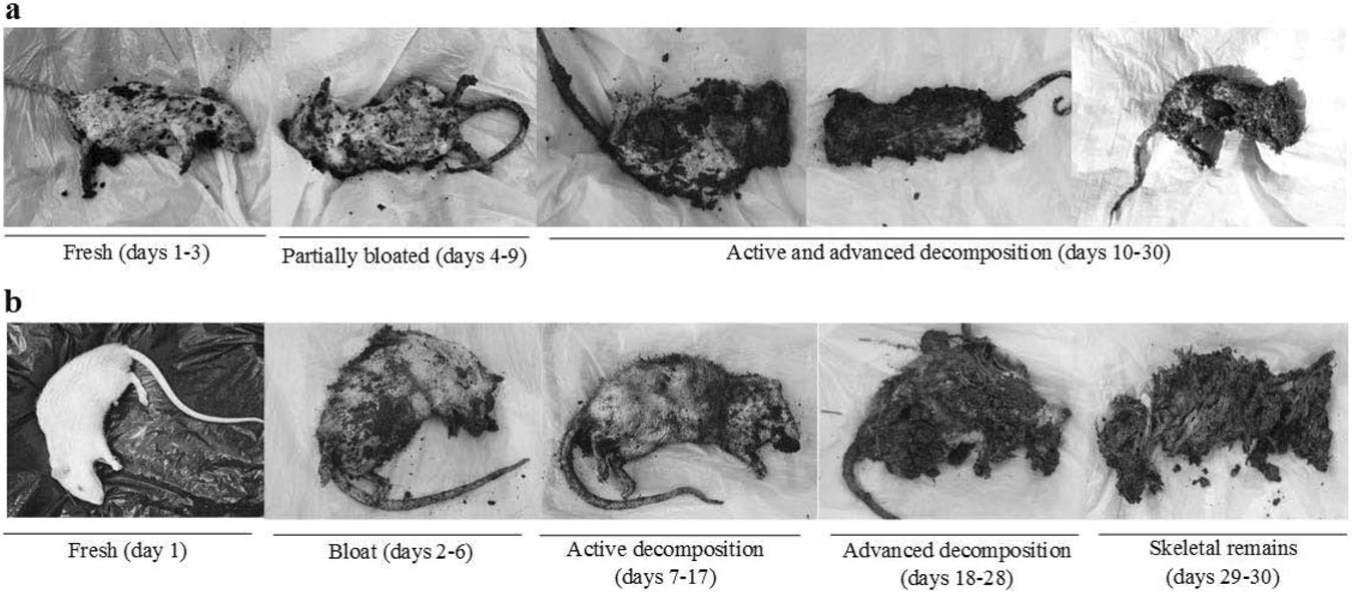
Decomposition stages of buried rat carcasses. (**a**) March; (**b**) June.

The overall decomposition process differed greatly between the two experimental periods (Fig. 2).

In March, given the low temperatures and high precipitation rate, the stages of decomposition were not clearly distinguished. The fresh stage covered days 1–3, followed by a partial swelling of the abdomen, with no visible discoloration in days 4–9. The active and advanced decay stages were not easy to distinguish, but detachment of epidermis and fur was observed on day 10. Day 18 marked the total detachment of fur and strong odour when opening the abdominal cavity. The organs of the abdominal cavity could be differentiated until day 25, having a brownish colour, and the small intestine was collected until day 30, the final day of the March experimental period (Fig. 2a).

The decomposition process in June could be differentiated into stages according to Wilson and collaborators^34^. The fresh stage was visible in day 1, followed by abdominal bloating, while the accumulation of gases was noticeable during days 2–6. The active decay stage (days 7–17) was marked by the deflation of the abdominal cavity, strong odour, with skin and flesh still present. Starting day 18, the advanced decay stage was represented by larvae present on the entire carcass surface, with most of the surface tissues very wet until day 25. Between days 25–30 most organs were liquefied, and the cecum decomposed. Still, portions of the small intestine could be collected until day 30 (Fig. 2b).

**Figure 3:**
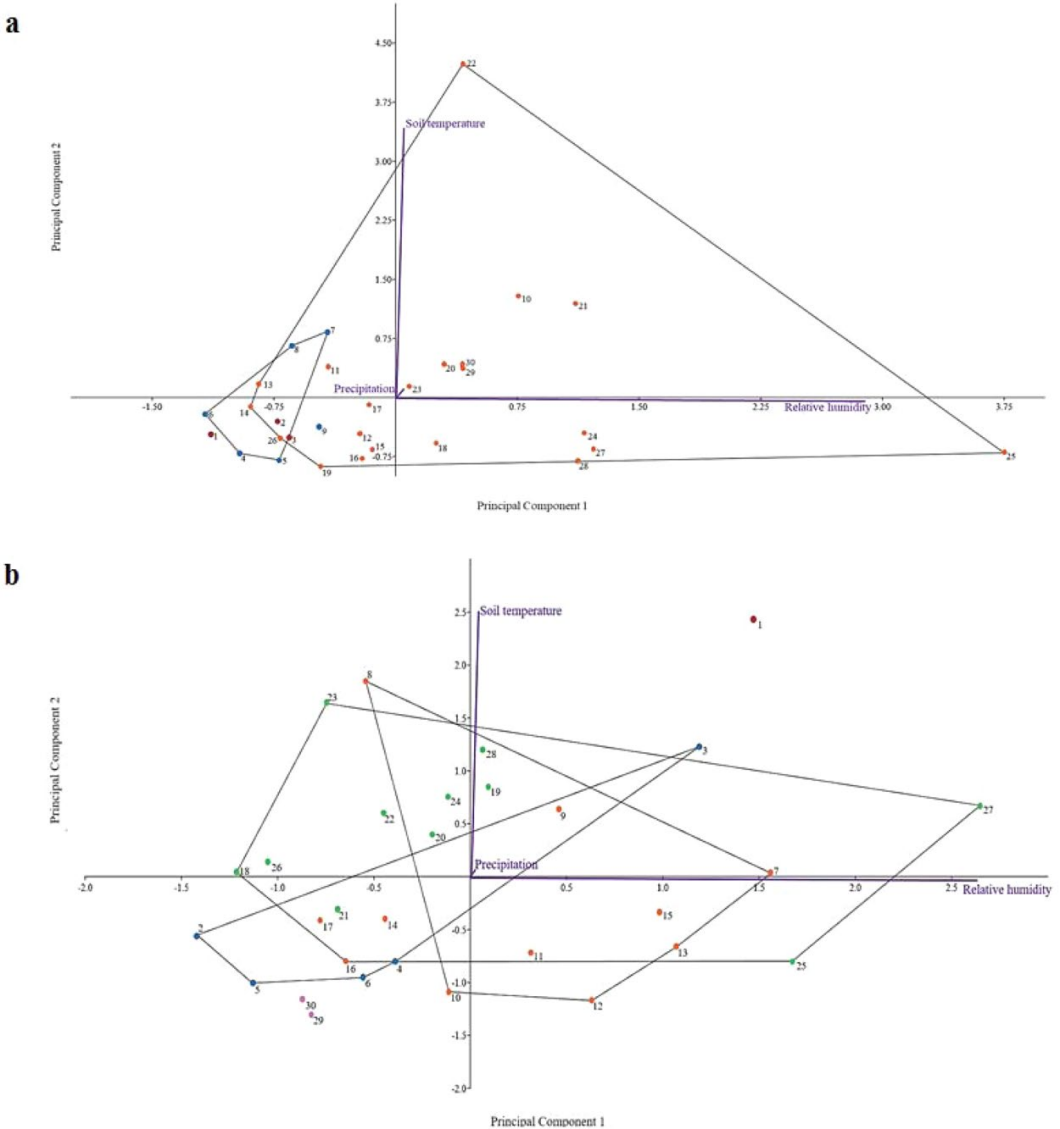
PCA of the environmental factors on the buried rat carcasses decomposition process during (**a**) March, and (**b**) June. 1–30 represent post burial days interval: fresh (red); partially bloated and bloated (blue); active and advanced decomposition (orange); advanced decomposition (green); skeletal remains (purple).

A PCA was used to investigate the impact of the soil temperature, relative humidity and precipitation rate on the decomposition process during March and June. The PCA used the decomposition stages as a grouping variable, while soil temperature, relative humidity and precipitation rate were used as independent variables. For March, the PCA explained 99.9% of the variance on the first two axes (PC1 and PC2), while the third axis only accounted for less than 1% of the total variance. The first principal component axis showed a high direct correlation with soil temperature, while the second principal component axis was highly correlated with the relative humidity (Fig. 3a). The PCA results were similar in June (Fig. 3b), where 99.9% of the variance was explained for the first two axes (PC1 and PC2) and less than 1% of the total variance was accounted for the third axis.

### Diptera and Coleoptera succession

**Figure 4:**
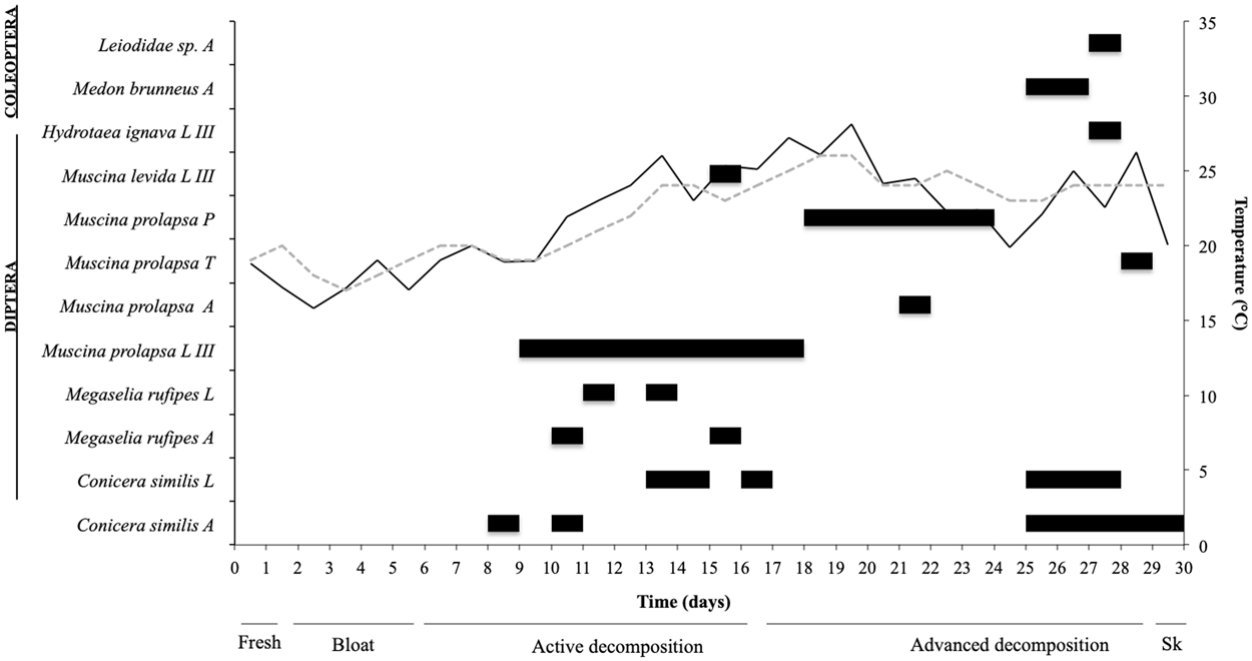
Diptera (black) and Coleoptera (grey) colonization pattern on rat carcasses, during June experimental period. Surrounding soil (grey dashed line) and air (black line) temperature variations and decomposition stages were indicated for the concerned period; A – adult; L – larvae; LIII – third instar larvae; P – pupae; T – teneral specimen.

No insect activity was observed during March due to low temperature records. In June, Diptera adults were observed during active decay stage (Fig. 4). The specimens were detected at the soil surface after 9 days’ post burial, though, no egg clusters were observed on the soil surface.

Three species belonging to Muscidae family were identified, *Muscina prolapsa* (Harris, 1780), *Muscina levida* (Harris, 1780), and *Hydrotaea ignava* (Harris, 1780). Only third instar larvae belonging to these three species were collected and identified. Starting with day 19, Diptera pupae were observed and collected, while adults’ emergence intervals were monitored. From all pupae collected only *M*. *prolapsa* emerged, and in most cases, 7 to 10 days passed until adult emergence. A teneral specimen of *M*. *prolapsa* was sampled from the rat carcasses during day 29. Also, two other Muscidae species were observed in the transition between active and advanced decay (*M*. *levida*) and at the end of advanced decay (*H*. *ignava*) (Fig. 4).

Two Phoridae species, *Megaselia rufipes* (Meigen, 1804) and *Conicera similis* (Haliday, 1833), both larvae and adults, were observed. The presence of *C*. *similis* was the first reported occurrence in Romania and now represents the species eastern most location^76^. *C*. *similis* adults were observed and sampled starting at day 9 and were not observed again until day 25 (Fig. 4).

Coleoptera adults (Staphylinidae and Leiodidae) were observed at the end of the advanced decay stage, starting with day 26. Staphylinidae species were identified as *Medon brunneus* (Erichson, 1839), and Leiodidae adult taxon was identified only at the family level (Fig. 4).

The taxonomic identification of both Diptera and Coleoptera specimens was confirmed by DNA barcoding. For Muscidae (Diptera), the COI amplicons of *M*. *prolapsa* third instar (584 bp) third instar, adult (621 bp) and teneral specimen (610 bp); and *M*. *levida* (615 bp), *H*. *ignava* (611 bp) third instars showed 100% identity with the GenBank reference sequences for *M*. *prolapsa* [KF919033.1], *M*. *levida* [KF919028.1] and *H*. *ignava* (as *leucostoma*) [JX861458.1], respectively. The COI amplicons of Phoridae (Diptera), *M*. *rufipes* adult (616 bp) and larvae (614 bp) presented 90% identity with the GenBank reference sequences for *M*. *rufipes* [KT111453.1] and [KP046535.1], respectively. Two Coleoptera adults belonging to Staphylinidae (623 bp) and Leiodidae (614 bp) showed 99% and 90% identity with the GenBank reference sequence for *M*. *brunneus* [KR487620.1] and Leiodidae sp. [KM843329.1], respectively.

### 16S rRNA Illumina MiSeq characterization of insect and rat small intestine tissues

#### Microbial community profile of necrophagous insect species

**Figure 5:**
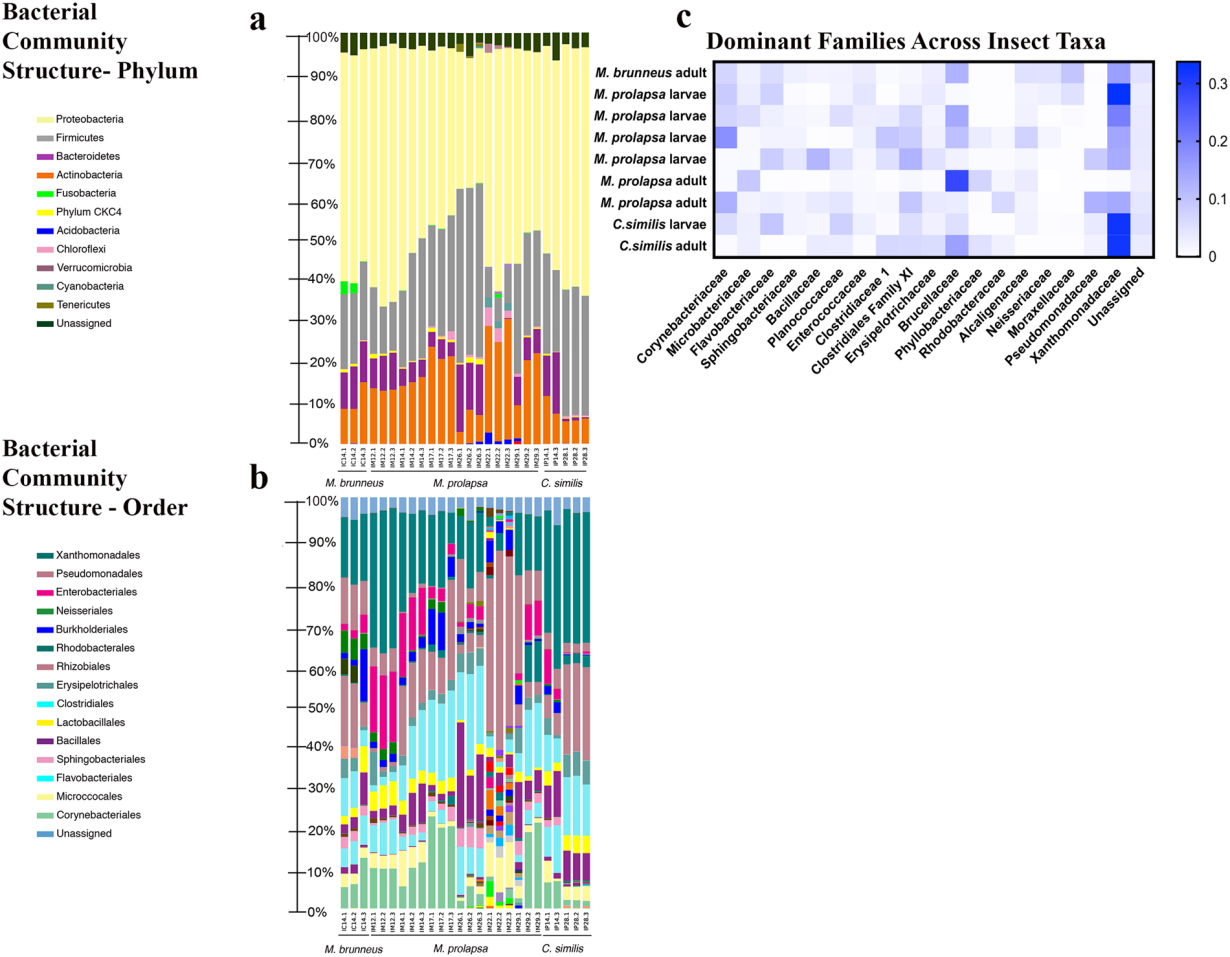
Bacterial community composition for insect-associated samples. (**a**) Phylum; (**b**) Order; (**c**) Family (Staphylinidae: *M*. *brunneus*; Muscidae: *M*. *prolapsa*; Phoridae: *C*. *similis*).

Bacterial communities associated with *M*. *prolapsa* third instar were characterized during days 12 and 14 which corresponded with active decay stage, day 17 at the transition between the active and advanced decay, and day 26 which corresponded with the end of the advanced decay stage. In addition, the bacterial community associated with *M*. *prolapsa* adults sampled during day 22 and teneral specimens collected on day 29 were analysed (Fig. 5). For the larval specimens, an increase of Firmicutes could be observed over the course of decomposition (13.13 (±2.58) % to 41.57 (±0.93) %), which also corresponded to a decrease of Proteobacteria (61.30 (±2.87) % to 32.50 (±0.92) %), especially during day 26 (Fig. 5a). For all insect specimens analysed, the dominant orders were represented by Xanthomonadales, Pseudomonadales and Clostridiales, while, Enterobacteriales, Bacillales and Corynebacteriales were observed in lower abundances (Fig. 5b).

The predominant families within phylum Firmicutes and Proteobacteria for *M*. *prolapsa* were Clostridiaceae and Xanthomonadaceae, respectively (Fig. 5c). The relative abundance of Firmicutes differed greatly between the teneral and adult *M*. *prolapsa* specimens showing an increase in abundance from 7.00 (±1.25) % in the teneral specimens to 24.97 (±1.76) % in the adult. The relative abundance of Proteobacteria however increased from the larval stages to 54.53(±3.87) % and 46.60(±4.96) % in the teneral and adult specimens, respectively. The relative abundance of Bacteroidetes, specifically families Flavobacteriaceae and Sphingobacteriaeae (Fig. 5c), did not vary greatly among larval samples between days 12–17 (5.53 (±2.13) %), until an increase during day 26 (13.37 (±2.67) %). Bacteroidetes variably differed for the adult and teneral specimens, being present in very low abundances in both adult specimens (<1.0% and 6.13 (±0.68) %, respectively). Actinobacteria, specifically families Corynebacteriaceae and Microbacteriaceae (Fig. 5c), exhibited an increase for the larval samples during the same period (days 12–17) (13.17 (±0.31) % to 21.87 (±1.53) %), with a considerable decrease in abundance on day 26 (5.90 (±2.65) %). In contrast, the teneral specimen exhibited the highest abundance of Actinobacteria (26.53 (±2.64) %), with the adult being lower (16.83 (±7.70) %).

The adult Coleoptera samples identified as *M*. *brunneus* were collected during day 25 (Fig. 5). Overall, *M*. *brunnes* had a similar microbial community to *M*. *prolapsa*. These adult microbial communities consisted primarily of Proteobacteria (54.20 (±2.25) %), Firmicutes (18.13 (±1.00) %), and Actinobacteria (10.63 (±3.78) %). However, adult *M*. *brunnes* had elevated abundances of Bacteroidetes compared to *M*. *prolapsa* (9.73 (±0.74) %).

Both larval and adult samples of *C*. *similis* were collected on days 14 and 28, respectively (Fig. 5). Similar, to the *M*. *prolapsa* specimens, the larval communities (day 14) consisted primarily of Proteobacteria (50.75 (±0.35) %), Firmicutes (22.10 (±3.25) %), and Actinobacteria (9.50 (±3.11) %)^76^. However, larval specimens of *C. similis* had higher abundance of Bacteroidetes (12.35 (±3.61) %) compared to *M*. *prolapsa* larvae (4.43 (±0.40) %).

#### Microbial community profile of the rat small intestine

**Figure 6:**
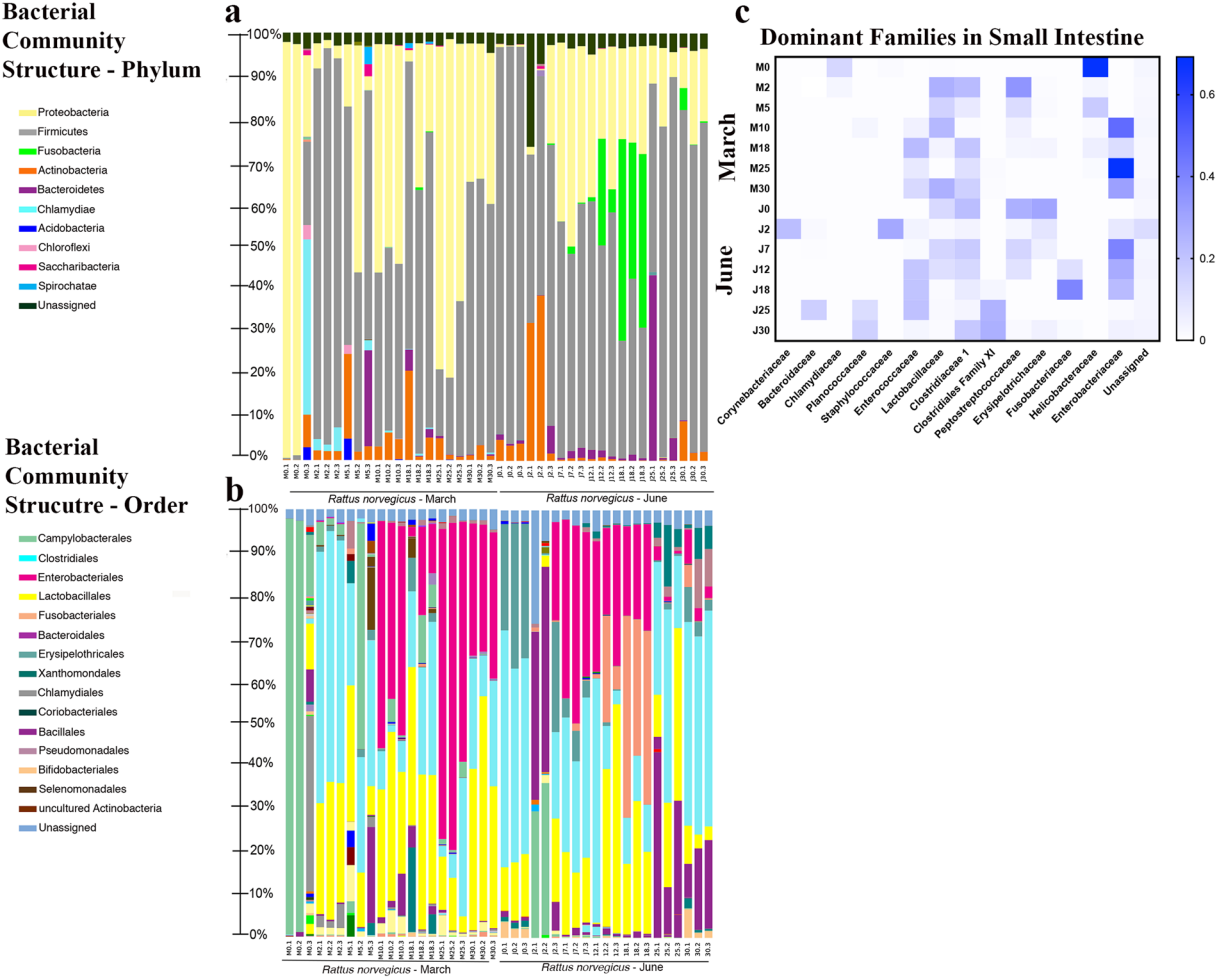
Bacterial community composition for rat intestine-associated samples. (**a**) Phylum; (**b**) Order; (**c**) Family (taxa representing ≥1.0% of the entire data set over time).

The first samples collected in March consisted of microbial communities with a high, but variable, abundance of Proteobacteria (70.87 (±44.83) %) and a low, but variable, abundance of Firmicutes (6.93 (±10.89) %) (see Supplementary Fig. S1). Immediately after day 2, an apparent shift between phyla occurred where Firmicutes increased and Proteobacteria decreased (88.57 (±3.59%) and 3.60 (±2.07) %, respectively). In the following days (5–10), a decrease of Firmicutes and an increase of Proteobacteria could be observed, which once again shifted on day 18. Towards the final part of the advanced decomposition stage, day 25, Proteobacteria predominated (71.61 (±10.04) %) but was then replaced by Firmicutes in the last experimentation day as the dominant phylum (33.10 (±1.95) %). Actinobacteria, Bacteroidetes and Acidobacteria were also present but never comprised more than 10% of the community (see Supplementary Fig. S1a). Overall, changes in the bacterial community relative abundance in March can be characterized by the apparent trade-off of phyla Firmicutes and Proteobacteria throughout the course of decomposition and the stable, but low, relative abundances of phyla Actinobacteria, Bacteroidetes, and Acidobacteria (Fig. 6a).

Unlike what was observed in March, the predominant taxa in the beginning of June belonged to Firmicutes (97.77 (±1.21) %) (see Supplementary Fig. S1b). Also, unlike what was observed in the beginning of March, early stages of decomposition (fresh and bloat) were characterized by the presence, though variable, of Actinobacteria (24.23 (±19.78) %) in June. Days 7 and 12 which corresponded with the active decomposition stage had similar abundance of Firmicutes and Proteobacteria to March samples, while Fusobacteria representatives were observed increasing in abundance throughout active decay reaching a relative abundance peak of (39.90 (±7.67) %) during advanced decay. Overall, changes in bacterial community relative abundance in June can be characterized by the dominance of phylum Firmicutes while other phyla decreased over time (Actinobacteria and Proteobacteria) or increased briefly but dissipated (Fusobacteria) (Fig. 6a).

At the order level, Clostridiales and Lactobacillales were more abundant in early decomposition in March, with Enterobacteriales being observed in the last part of the decay process (Fig. 6b). Clostridiales and Lactobacillales were also abundant in first part of June, along with Xanthomonadales. During advanced decay stage, an increase of Fusobacteriales was apparent, while Bacteroidiales increased in abundance through the end of decomposition (Fig. 6b). During the experimental period, the most abundant families belonged to Helicobacteraceae, Enterobacteriaceae, and Peptostreptococcacae (Fig. 6c).

#### Overall diversity of insect and rat intestine microbial communities

**Figure 7:**
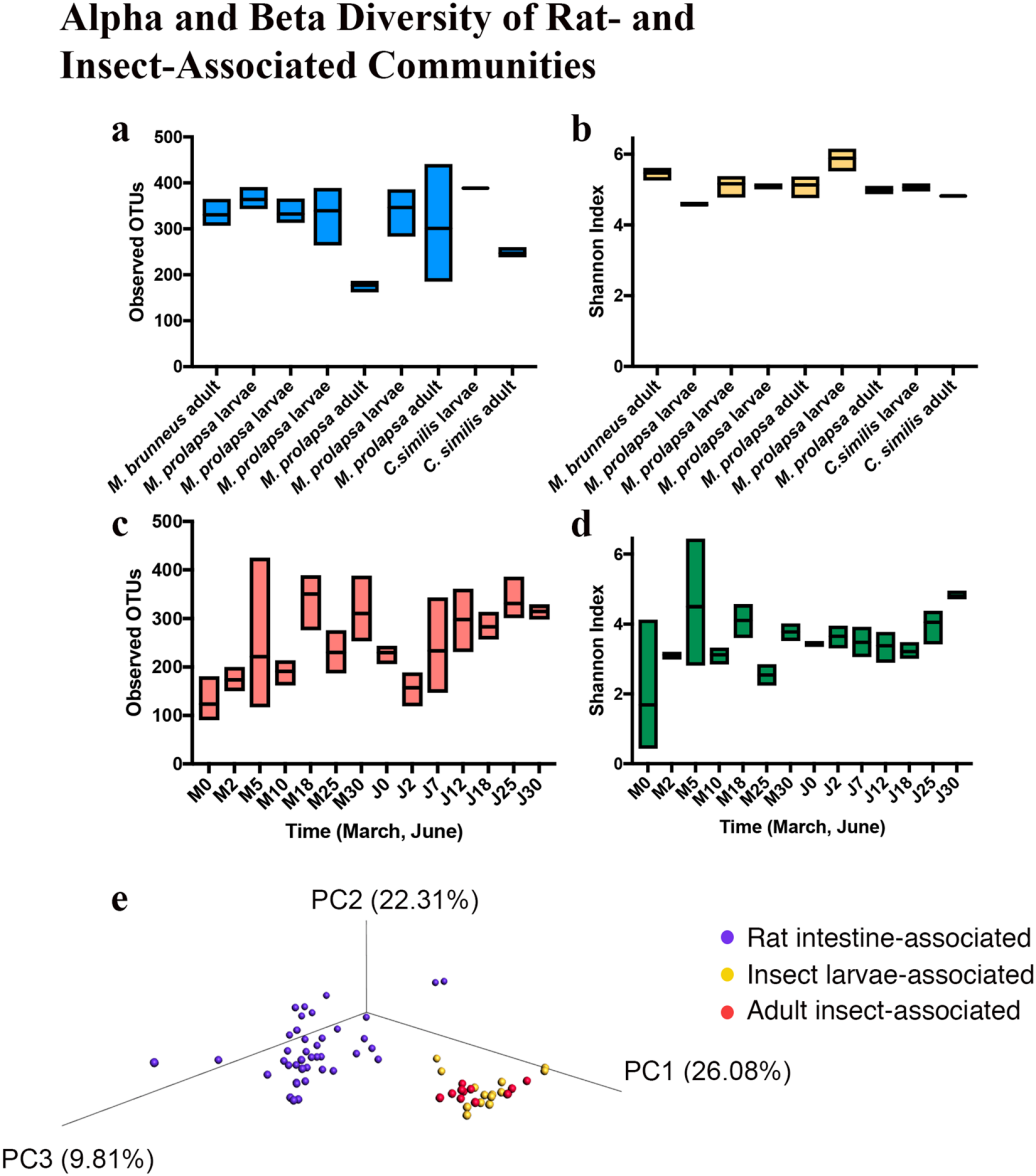
Alpha diversity represented by OTU richness and Shannon’s Diversity Index for insect–associated (**a**,**b**), and rat intestine–associated (**c**,**d**). Beta diversity of decomposition microbiome based on weighted UniFrac distances (**e**).

The alpha diversity of insect larval samples had higher richness (Fig. 7a) compared to the adult samples. Further, the alpha diversity for the insect communities had higher richness (Fig. 7a) and diversity (Fig. 7b) compared to the rat intestine (Fig. 7c,d). Not only did communities differ within samples, bacterial communities were different between samples (Fig. 7e). The total diversity of taxa was similar between the two experimental periods. For March 23 phyla, 103 orders, and 410 genera, were identified, while for June 23 phyla, 95 orders, and 374 genera were identified. To explain some of the variation seen among microbial communities a Pearson’s correlation analysis with the pH and Shannon diversity was run but showed that there was no significant correlation between the bacterial diversity and pH (*p* = 0.41, *r2* = 0.06).

### *Enterococcus faecalis* and *Clostridium paraputrificum* postmortem relative abundances

**Figure 8:**
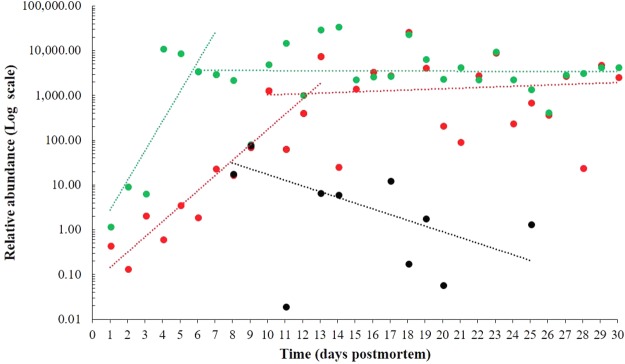
Relative abundance of Firmicutes species as a function of the postmortem time. *Enterococcus faecalis* (March – red; June – green), *Clostridium paraputrificum* (March – black). Logarithmic scale representing number of bacterial cells.

Since phylum Firmicutes composes a major portion of the human gut microbiome^50^ and are frequently identified during human decomposition, *Enterococcus faecalis* and *Clostridium paraputrificum* were selected for quantitative analysis to determine their postmortem relative abundance with the intention of identifying putative PMI microbial markers (Fig. 8).

For *E*. *faecalis* (Fig. 8), the abundance varied between the March and June experiments, where the highest bacterial content was reached at different decomposition stages, corresponding to 10^2.5^ for active decay and 10^4.5^ for advanced decay. In March, a logarithmic increase in bacteria was observed from the fresh stage to the initial active decomposition stage (days 1–13), that became consistent throughout the rest of the experimental period (days 13–30). In June, a rapid increase in *E*. *faecalis* abundance was observed during the fresh and bloat stage (days 1–6), that quickly reached a plateau during the start of active decomposition (days 7–30), with small variations.

Although the initial content of *E*. *faecalis* in rat intestine was about 5-fold lower during March experiment than June, the changes in abundance of this species towards the final period of carcass decomposition was similar (10^3.5^ ± 10^2.19^). However, the growth of this bacterium during initial decomposition stages appeared to be strongly dependent on the environment (soil temperature).

*C*. *paraputrificum* was detected during March beginning with day 8 postmortem, which corresponded to active and advanced decomposition stages (Fig. 8). The relative abundance of this species (10^1.1^) was about 200-fold lower than that of *E*. *faecalis* (10^3.4^) and showed an overall decrease during the decomposition process. During June, the presence of this species was sparse, and a specific trend could not be established. The presence was observed only during the active decay stage, corresponding to days 11 and 15 postmortem.

## Discussion

Decomposition of buried remains, whether human^20–23^ or non-human mammal^13,14,18^ is less studied compared to surface remains, especially regarding research on necrophagous insect species and necrobiome. The present study provides an insight on buried carcass decomposition at 40 cm depth for postmortem interval estimation.

### Decomposition dynamics

The depositional environment encompasses environmental parameters like temperature and soil chemistry, that can influence the decomposition process^80^. Further, the amount of oxygen available in the soil based on soil type, can influence the rate of decomposition and even the structure of the microbiological environment^81^. As such, if buried carcasses experience lower temperatures and different environmental conditions from contact with the soil, differentiating buried and exposed taphonomic studies, especially in the context of PMI estimation, becomes important^30^. Temperature is a significant factor influencing decomposition, with the optimal values for soft tissue decomposition ranging from 20 °C to 40 °C^82^. Throughout the present experiment, temperature played an important role from two points of view. First, the lower temperature values recorded during the early spring experiment (March) inhibited the putrefaction process. During that time, the soil and air temperatures varied between 7.28 °C–10.76 °C and 3.96 °C–16.3 °C, respectively, and the carcass decomposition was not completed by the end of day 30.

Secondly, the stages and their transitions were not clearly differentiated, and the lower temperatures thresholds could have prevented insect species presence and activity, considering that temperatures above 10 °C and 15 °C are optimum for their activity^83^. Unlike March, June was characterized by a soil and air temperature of 16.75 °C–25.37 °C and 15.8 °C–28.13 °C, respectively. The separate decomposition stages were observed entirely up to the beginning of skeletonization. It is of note that the minimum soil temperatures in both experimental periods were higher than the air temperatures, with the highest values observed above ground. Though, the temperature doesn’t fluctuate very much in soil, lower thresholds can prevent, inhibit, or stop decomposition^84^, similar to what was observed during March of the current study.

Further, temperature plays a critical role, among others, in estimating the postmortem interval^26^. For exposed conditions, meteorological data can be obtained from weather stations, but acquiring underground data is difficult. Luckily, temperature fluctuations are reduced compared with the above ground conditions^30^, and there were no significant differences between air and soil temperature in our experiment, with a minimum difference of 0.04 °C and a maximum of 5 °C recorded in March. In June, the maximum difference between air and soil temperature was of 2.89 °C. This small difference between the air and soil temperature records was also observed in previous studies^85^. During forensic investigations air temperature records can be used as an alternative in the absence of soil temperature data for postmortem interval estimation. However, the interpretation of such results must be performed with caution, as these data are often variable and dependent on climate and soil type.

Lastly, temperature can influence bacterial proliferation and diversity. Studies have shown that lower temperatures can inhibit the microbial activity leading to the decrease in rate of decomposition and the preservation of a body, while temperatures ranging between 10 °C and 30 °C can accelerate the microbial activity, specifically the production of enzymes responsible for the decomposition of organic matter^12^.

Though we cannot compare our bacterial results to an exposed carcass, the soil temperature and ambient temperature did not differ with average difference of only 5 °C indicating that temperature alone would not have solely affected the microbial community.

The soil chemistry of the depositional environment, like the pH, can also affect the microbial community structure over time. For instance, Turner and Wilshire^86^ reported that a very high alkaline soil may inhibit the survival of bacterial taxa involved in decomposition. However, in the current experiment, the soil was slightly alkaline (max. pH 8.84 in March), thus not affecting the changes of bacterial abundance, as demonstrated by the lack of association in the Pearson’s correlation analysis. Also, Forbes^36^ indicated that a slightly alkaline soil can favour bacteria involved in decomposition to thrive.

In both March and June, the overall increase in pH was observed from early decomposition in March and beginning of active decay in June. This trend differs from what Carter and Tibbett^12^ observed where the soil alkalinity was high in the early phases, followed by a decrease in pH (4.7). Further, another experiment by Carter and collaborators^19^ on juvenile rat specimens demonstrated an increase of soil pH up to 8.0 in all grave soils, consistent with our current findings. Also, Wilson and collaborators^34^ witnessed a significant increase in pH during the pig carcasses decomposition process, at depths of 0.3 and 0.6 cm. However, the soil type and moisture must be taken into consideration as it could influence any similarities or dissimilarities among these studies, including oxygen content, though no such data was collected in the present experiment.

### Insect dynamics on buried rat carcasses

The depositional environment can also encompass biological factors such as insect colonizers and microbial communities^87^. The occurrence of Diptera and Coleoptera specimens was recorded during June experimental period, starting with day 9 post burial, at temperatures of 18.67 °C.

Our findings with *M*. *prolapsa* are largely consistent with previous studies in terms of the depth at which *M*. *prolapsa* can colonize and its peak activity seasons. First, *M*. *prolapsa* was able to colonize at a depth of 40 cm, behaviour observed also during Gunn and Bird experiment^13^, even when bait was present at the soil surface. However, that study showed that adult Muscidae would colonize buried remains in 30 min when enclosed^13^. We observed colonization after 9 days. Even though *M*. *prolapsa* was observed colonizing buried remains in both cases, the exposure conditions could make a difference in colonization timing since the current study did not enclose or restrict insect movement. Also, in a previous study that used rat carcasses buried form 10–40 cm, *M*. *prolapsa* was observed to colonize and eventually pupariate after 8–12 days^28^. We observed a similar pupation time of 10 days with rats buried at 40 cm. Only *M*. *prolapsa* puparia were collected at 40 cm unlike Gunn and Bird^13^, where they were observed in the first 4 cm of soil.

Lastly, *M*. *prolapsa* was shown in the UK to be active in June-August^14^, similar to what we observed in Romania in June. Usually, *M*. *prolapsa* is encountered during the warmest months of the year in countries that have a temperate climate^14^. Even though the activity period was broader in other experiments^28,88,89^, covering early spring to late autumn, June and July were generally accepted as the two primarily activity peaks for this species. This behaviour was also observed throughout this experiment, though not surprising given this species’ cosmopolitan presence in this eastern part of Europe and Romania’s temperate climate conditions. Another Muscidae species, *M*. *levida*, was identified in both the previous^13^ and the current study, though being observed only briefly. Overall, *M*. *prolapsa* can be considered an indicator of postmortem interval estimation for buried remains, due to the species’ ability to detect and colonize buried remains, its prevalence under these conditions^28^, and its cosmopolitan occurrence^14^.

Phoridae represented the second family of Diptera identified during the June experiment. The species within this family are representatives for buried environments, being observed from 10 cm down to 2 m (coffin depth)^83,90,91^. Mostly, *Conicera* species have been observed, collected and identified during previous experiments^86,92^. The current data observed two Phoridae species, *Conicera similis* and *Megaselia rufipes*, with *Conicera* being more abundant and consistently observed^76^. In the current case *C*. *similis* adults were observed after 9 days post burial, while previous studies reported *Conicera* sp. after 4 days post burial and at a depth of 25–50 cm^93^. *M*. *rufipes* adults and larvae were observed and collected during days 11–16, corresponding to the middle and end of the active decomposition stage, similar with what has been reported so far for the sister species, *M*. *scalaris* Loew 1866^85^, on buried pig carcasses at 60 cm.

Staphylinidae (Coleoptera) representatives exhibit predatory behaviour towards dipteran immature stages and are often encountered during the last stages of decomposition^30^. Though, this family is less dominant in soil, it is often observed in burial environments^30^. Other studies^24,94,95^ have reported *Philonthus* sp. as a prominent species recovered from buried carcasses, though we observed only *M*. *brunneus* at the end of the advanced decomposition stage. Nevertheless, coleopteran species are not used for postmortem interval estimation and not considered taxa of forensic interest^30^. Their presence is only noted due to the predatory behaviour, which can affect the dipteran larval masses and the overall decomposer community.

### Insect microbiome

To better understand the bacterial communities associated to necrophagous insect adults and larvae, three species were analysed: *M*. *brunneus*, *M*. *prolapsa*, and *C*. *similis*. A more in-depth analysis of *C*. *similis* can be found in a previous paper and will not be discussed here^76^.

A previous study investigated the insect-associated bacterial communities during the development process of two Calliphoridae species (*Lucilia sericata* Meigen, 1826 and *Lucilia cuprina* Wiedemann, 1830)^96^. Even though their investigation concerned a different necrophagous Diptera family, Proteobacteria and Firmicutes were dominant phyla, similar to the current observations of Diptera here and more recent experiment with other Calliphoridae^49^. Unlike previous studies, the current study shows that Actinobacteria was an abundant phylum with relative abundances of 15–25% across insect taxa. This could be due to both a difference in insect taxa and laboratory versus *in situ* experiments where our samples were in contact with the soil. Overall, species belonging to genera *Ignatzshineria*, *Proteus* and *Clostridium* were common regardless of the necrophagous insect family. As previous experiments show^96–98^, certain bacterial taxa are associated with insect species, at times producing enzymes that help breaking down the tissues in the decomposition process.

### Carcass microbiome

Several studies have investigated the microbiome of decomposition. Overall, these studies show a decrease in microbial diversity relative to a decrease in nutrients until skeletonization^99^, something not observed in the present data as diversity remained variable but stable over time. However, the majority of these experiments used exposed carcasses of mice^11,45,48^, humans^11,41,44,47,100,101^, or swine^31,40^. An exposed carcass can differ in many ways including the depositional environment and the decomposer communities and colonizers involved in decomposition, both of which can impact the rate of decomposition and PMI estimation^29^.

The current study is similar to previous studies regarding the decomposition of the intestines and abdominal cavities largely associated with trade-off like changes between phyla Firmicutes and Proteobacteria^42,48^. However, unlike either study, we observed a lesser overall abundance of Bacteroidetes and an increased abundance of phylum Actinobacteria. First, it has been shown that living rat gut microbiomes have less abundance of Bacteroidetes compared to living human gut^102,103^ which could explain starting differences post mortem between studies. Second, the Actinobacteria are common soil bacteria^104^. Since rat carcasses were buried, these could be soil-associated microbes though the soil microbial communities were not characterized in the present study. It is also important to note here that the lack of an exposed carcass as a burial control doesn’t allow for resolution of differences between insect or microbial community dynamics of buried or exposed remains, but rather provides a descriptive observation of buried rat carcasses. Nevertheless, Firmicutes was most abundant during the first postmortem day during June, similar to previous studies on decomposed rat carcasses^45^. Though in March, Proteobacteria represented the dominant phylum. These differences in phyla abundances may be a result of temperature. As Firmicutes was most abundant at higher temperatures both in a previous study^45^ and the present experiment, while Proteobacteria was most abundant during lower temperatures recorded during March.

It is important to note that mammalian host microbial community structure and composition exhibit both interspecies variation^105^ and intraspecies variation, as in the case with humans. This highlights an increasingly important point for the future of decomposition studies in that the microbial communities among the host, the environment, and the colonizers and scavengers could all have potential effects on the community structure and dynamics, and thus on the estimation of postmortem interval.

### In search of bacterial PMI biomarkers

During March and June two Firmicutes taxa (*Enterococcus faecalis* and *Clostridium paraputrificum*) were quantitatively analysed during the decomposition of buried rat carcasses, representing the first analysis of its kind concerning these taxa.

*E*. *faecalis* is a known commensal bacterial species of the human and non-human mammal gastrointestinal tract^106,107^. It can be an opportunistic pathogen responsible for nosocomial infections, often identified in small numbers in healthy human small intestine^108,109^, where it survives mainly by fermentation of non-absorbed sugars^110^. Moreover, *E*. *faecalis* can survive in very harsh conditions (salinity and pH) and can catabolize a wide range of resources^111^.

Given this taxon’s capacity to ferment glucose and to catabolize carbohydrates, diamino acids and glycerol^112^, the exponential increase in relative abundance during the bloat stage in both experimental periods, was not surprising as the carcass is being rapidly consumed and microbes are proliferating.

While the initial relative abundance of this species is different depending on the season, the abundance of *E*. *faecalis* in March and June become more similar as decomposition progresses. The observed increase in abundance in *E*. *faecalis* in June can be explained by the temperature difference between March and June when the summer month had higher abundance records of this species. This is consistent with summer temperature being closer to the optimal temperature for *E*. *facaelis* of 37 °C, though this genus can grow in a broad range of 10 °C to 42 °C^113^.

The potential use of this species as an indicator of the PMI is promising, given the likely presence in intestinal tissues^114^. However, this hypothesis requires future research, since the composition of the bacterial communities varies between, within and across mammalian gastrointestinal tracts and could be influenced by manner of death.

*C*. *paraputrificum* was quantitatively analysed from the same samples and its presence was first observed at the transition between partially bloated and active decay. In contrast to *E*. *faecalis*, this taxon is infrequently encountered during human infections, and its clinical significance is not well described^115^. Studies addressing the quantification of Clostridiales involved in decomposition process have been performed only at the family level, without targeting a certain species^101^. This bacterial species is known to be strictly anaerobic, chitinolytic, Gram-positive and mesophilic, producing acetic and propionic acids, and large amounts of gas from the fermentation process^116^. Though this type of metabolism seems to coincide with the bloat and active decay as microbial biomass is producing much gas, this taxon was not consistently observed, and a determination could not be made. Given the fact that this taxon is less studied^117^ and no information is available regarding the role played in decomposition, future investigations are needed.

Currently, there are few studies focusing on the quantification of decomposition-associated bacteria to be used as putative markers for PMI estimation, especially related to phyla Firmicutes^50,101^, or Actinobacteria and Bacteroidetes representatives. Our study showed an increase of the relative abundance of the *E*. *faecalis* (Firmicutes) with 3–4 orders of magnitude, while Hauther and collaborators^50^ observed that the relative abundance of other Firmicutes taxa (*Lactobacillus spp*.) declined with three orders of magnitude. Overall, these data show that it is very plausible to use quantitative microbiome information for a potential postmortem interval estimator. However, one must expect quantitative differences as each carcass has its own specific ante mortem microbiome composition.

The possibility of targeting shared or “core” bacterial taxa and describing their relative abundances ante and post mortem, represents potential postmortem interval estimators.

Complementing entomology with microbiology will strengthen the current knowledge regarding decomposition of buried carcasses by providing a holistic view of the decomposition process to be used during forensic experiments and investigations.

## Electronic supplementary material

Supplementary Figure 1

## Data Availability

The raw 16S rRNA datasets generated during the current study and are available from the corresponding author on request.
