# Supplementary material for: Characterizing forensically important insect and microbial community colonization patterns in buried remains
Source: Sci Rep. 2018 Oct 19;8:15513. doi: 10.1038/s41598-018-33794-0 (PMC6195615; doi:10.1038/s41598-018-33794-0)
Supplement: Supplementary file 1 — Supplementary Figure 1 [file 41598_2018_33794_MOESM1_ESM.docx]

**Characterizing forensically important insect and microbial community colonization patterns in buried remains**

**Lavinia Iancu^1^***, **Emily N. Junkins^2^**, **Georgiana Necula-Petrareanu^1^**, **Cristina Purcarea^1^**

^1^Institute of Biology Bucharest, Romanian Academy, Splaiul Independentei, 296, 060031, Bucharest, Romania

^2^University of Oklahoma, Department of Microbiology and Plant Biology, 770 Van Vleet Oval, Norman, OK 73019-0390, United States of America

*** Corresponding author:** [lavinia.iancu@outlook.com](mailto:lavinia.iancu@outlook.com)


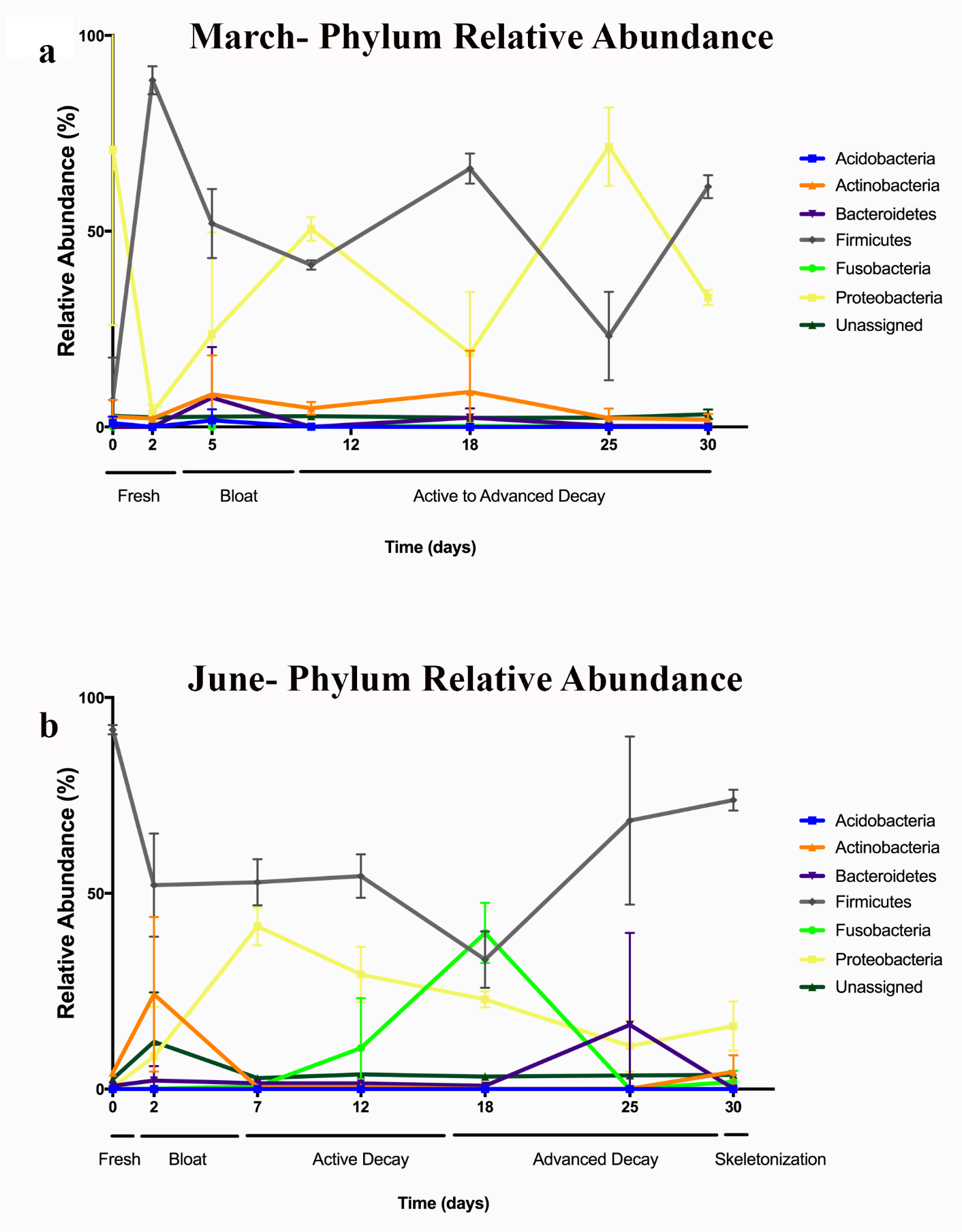


**Supplementary Fig.1**. Bacterial relative abundance change overtime for rat intestinal communities: (a) March; and (b) June. Standard deviations indicated by error bars (*n*=3).
